# 

**Published:** 1891-10-03

**Authors:** 


PRICE ONE PENNY.

No. 262, Vol. XI.—October 3rd, 1891.

[Registered at the General Post Office as a Newspaper for transmission in the United Kingdom and Abroad.]

WITH EXTRA NURSING SUPPLEMENT.

Per Annum, 6s. 6d.; post free.

Monthly Parts, 6d.; post free 8d.

Ditto per Annum, 8s., post free.

### THE HOSPITAL

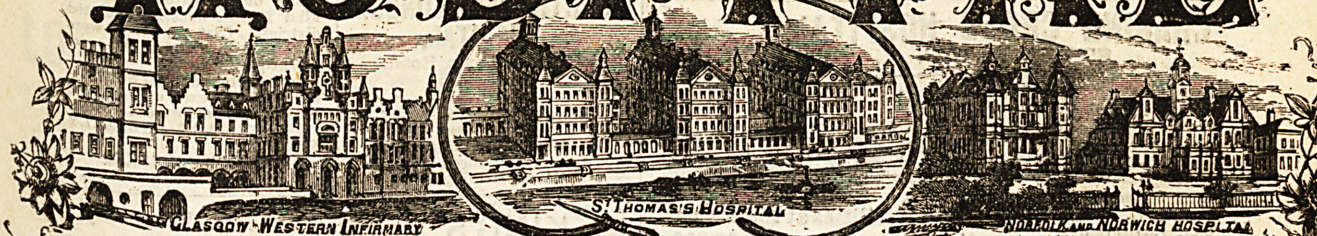

A WEEKLY JOURNAL OF

Science, Medicine, Nursing and Philanthropy.

### SATURDAY, OCTOBER 3rd, 1891.

**Peace or War?** . . . . . . . . . . . . 1

**Passing Topics.**—Shall Nurses Cease to be Laywomen?—IV. . . . . . . . . . . . 2

**The Doctor's Armchair.**—Ouida against the Physiologists 3

**The History and Capabilities of Herbal Simples.**—By W. T. Fernie, M.D., XXXVII.—Ferns (*continued*) . 4

**Editor's Letter-Box.**—Treatment of Scarlet Fever and Diphtheria by the Diniodide of Mercury—Dr. Collie Again . 5

**Notes and News** . . . . . . . . . . 6

**Annotations.**—African Philanthropy and Dividends—Animated Water at Birmingham . . . . . . 8

**General Charities**—Scraps and Gleanings . . . 7

**The Practitioner's Mirror and Retrospect**—

Medicine—New Methods of Treatment in Phthisis I.—The Roussel Treatment . . . . . . . . . 9

Surgery—Genu Valgum.—II. . . . . . . . 10

Therapeutics—Salol in Typhoid Fever . . . . . 11

New Drugs, Appliances, and Things Medical—At the British Medical Association (continued) . . . . . 11

**Poisons and Poisoners.** III.—Antimony: Dr. Pritchard . 12

**The Student of Medicine.**—Appointments—Vacancies—Pass Lists

### EXTRA SUPPLEMENT—THE NURSING MIRROR.

**En Passant.**—Lowestoft Nurses' Association—Halifax Union—Short Items—The Lay Press and the Nurses—Nurses for the Middle Class—Male Nurses . . . . . . i

**The Cyprus Society**—Educational Work . . . . ii

**Hints to Nurses.** By Wm. J. Rugg, M.R.C.S.Eng., L.R.C.P. Lond. . . . . . . . . . . . ii

**Another Letter on India** . . . . . . . . iii

**For Reading to the Sick**—The Husbandman . . . . iii

**Notes from Australia** . . . . . . . iv

**Presentations** . . . . . . . . . . iv

**Everybody's Opinion.**—Religion and Disease—Nurses and Students—The London Hospital—Nurses' Food . . . . v

**Where to Go** . . . . . . . . . . v

**Wants and Workers** . . . . . . . . v

**Notes and Queries** . . . . . . . . v

**Off Duty.**—Kitty's Find—Strayed Away . . . . . vi

**Appointments** . . . . . . . . . . vi

**Amusements and Relaxation** . . . . . . vi

